# Supplementary material for: TGFβ signaling directs serrated adenomas to the mesenchymal colorectal cancer subtype
Source: EMBO Mol Med. 2016 May 24;8(7):745–60. doi: 10.15252/emmm.201606184 (PMC4931289; doi:10.15252/emmm.201606184)
Supplement: Supplementary file 1 — Appendix [file EMMM-8-745-s001.pdf]

TGFβ signaling directs serrated adenomas to the mesenchymal colorectal cancer subtype

Appendix

Table of contents

Appendix supplementary materials and methods

TA organoid culture

Normal colon organoid isolation and culture

Quantitative real-time PCR primer sequences

CIMP, *BRAF*, and *KRAS* mutation status analysis in patient-derived adenoma samples

Appendix figures

Appendix Figure S1

Appendix Figure S2

Appendix Figure S3

Appendix supplementary materials and methods

TA organoid culture

TA organoids were cultured in TA culture medium: advanced DMEM/F12 (Gibco), supplemented with N2 and B27 supplement (Invitrogen), penicillin/streptomycin (PS), gentamycin, amphotericin B, 2 mM L-glutamine, 0.15% D-glucose (Sigma), 100 µM β-mercaptoethanol (Sigma), trace elements B and C (Fisher Scientific), 5 mM HEPES (Life Technologies), 2 µg/ml heparin (Sigma), 10 µg/ml insulin (Sigma), 20 ng/ml human EGF (PeproTech), and 10 µM SB202190 (Sigma). Organoids were grown in matrigel and were disrupted mechanically every 7 days. Medium was refreshed 3 or 4 days after dissociation.

Normal colon organoid isolation and culture

Normal colon organoid cultures were isolated as previously described (Sato et al, 2011). In brief, the muscle layer and submucosa were removed from normal colon tissue and the mucosa was washed in PBS + PS + gentamycin + amphotericin B (further referred to as PBS + antibiotics) for 15 minutes at RT. The tissue was cut in small pieces and incubated in PBS + antibiotics + 10 mM DTT (Sigma) 3 times, each 5 minutes at RT. Subsequently, the tissue was transferred to PBS + antibiotics + 8 mM EDTA (Invitrogen) and incubated with slow rotation at 4°C for 1h. After the incubation period, the supernatant was removed and replaced with PBS + antibiotics. Through thorough shaking, crypts were removed from the tissue and the supernatant containing the crypts was transferred to a new tube. The procedure was repeated three times and the obtained crypts were pooled, FBS was added to a final concentration of 5%, and the

crypts were pelleted by centrifugation at 600 rpm for 2 minutes. The supernatant was removed and the crypts were washed three times in advanced DMEM/F12 + 2 mM GlutaMAX-1 (Invitrogen) + 10 mM HEPES + 5% FBS + PS + gentamycin + amphotericin B. Lastly, the crypts were resuspended in matrigel and plated in 24 well plates. Normal colon organoids were dissociated every 7-10 days, every 3-4 days medium was refreshed. Normal colon culture medium: advanced DMEM/F12, supplemented with N2 and B27 supplement, PS, gentamycin, amphotericin B, 2 mM GlutaMax-1, 10 mM HEPES, 1 mM N-acety-L-cysteine (Sigma), 10 nM [Leu15]-gastrin I (Sigma), 10 mM nicotinamide (Sigma), 500 nM A83-01 (Tocris), 3 μM SB202190 (Sigma), 50% WNT3A conditioned medium, 50 ng/ml h-EGF, 20% RSPO1 conditioned medium, 10% Noggin conditioned medium, 10 nM PGE2 (Santa Cruz Biotechnology).

Quantitative real-time PCR primer sequences

Primer sequences: *ACTB*-forward: 5'- CAG AAG GAT TCC TAT GTG GGC GA; *ACTB*-reverse: 5'- TTC TCC ATG TCG TCC CAG TTG GT. *ZEB1*-forward: 5'- GCA CAA GAA GAG CCA CAA GTA; *ZEB1*-reverse: 5'- GCA AGA CAA GTT CAA GGG TTC. *FRMD6*-forward: 5'- TGC TCA GGC TAA AGG ACT GC; *FRMD6*-reverse: 5'- GTA CTG CAC ACG GAA GTG GA. *CDX2*-forward: 5'- TTC ACT ACA GTC GCT ACA TCA CC; *CDX2*-reverse: 5'- TTG TTG ATT TTC CTC TCC TTT GC. *BCL2L11*-forward: 5'- TAA GTT CTG AGT GTG ACC GAG A; *BCL2L11*-reverse: 5'- GCT CTG TCT GTA GGG AGG TAG G. *BID*-forward: 5'- CAT CCC TCC GGG CCT GGT GA. *BID*-reverse: 5'- AGG GTA GGC CTG CAG CAG CT. *BBC3*-forward: 5'- GAC GAC CTC AAC GCA CAG TA;

BBC3-reverse: 5'- GTA AGG GCA GGA GTC CCA T. *KI-67*-forward: 5'- GCC TGC TCG ACC CTA CAG A; *KI-67*-reverse: 5'- GCT TGT CAA CTG CGG TTG C.

CIMP, *BRAF*, and *KRAS* mutation status analysis in patient-derived adenoma samples

For patient-derived adenoma samples TA8, TA9, TA10, TA11, TA12, TA13, SSA2, SSA3, SSA4, SSA5, SSA6, SSA13, and SSA14 the *BRAF* and *KRAS* mutation status was determined using a multiplex PCR as described in the materials an methods section "Mutation analysis" in the main text (Lurkin et al, 2010). For all other patient-derived adenoma samples the *KRAS* and *BRAF* mutation status were analyzed as described below.

KRAS mutation status of codon 12, 13, and 61 was determined by amplification of exon 2 and exon 3 of the *KRAS* gene and subsequent Sanger sequencing (see the materials and methods section "Mutation analysis" in the main text for the protocol and primer sequences of *KRAS*_exon2). Primer sequences: *KRAS*_exon3-forward: 5'- CAG GAA GCA AGT AGT AAT TGA TGG; *KRAS*_exon3-reverse: 5'- TGG TGA ATA TCT TCA AAT GAT TTA GT.

BRAF mutation status was determined by quantitative real-time PCR in a 10 µl total reaction volume consisting of 20 ng gDNA or cDNA, 5 µl SYBR Green (Roche), 1 µl forward and reverse primers (10 µM), and 3 µl H2O. Each sample was measured in triplicate for each primer pair on a Roche Light Cycler 480 II in accordance with the manufacturer's instructions (annealing temperature of 60°C). gDNA or cDNA of a $BRAF^{wildtype}$ and a $BRAF^{V600E}$-mutated cell line were included in each run as control. The

resulting Cp-values of the mutant primer pair was subtracted from the wildtype one. Samples were scored as *BRAF*^{V600E}-mutant if this difference was < 5. Primer sequences: *BRAF*^{wildtype}-forward: 5'-AGG TGA TTT TGG TCT AGC TAC AGT, *BRAF*^{V600E}-forward: 5'- AGG TGA TTT TGG TCT AGC TAC AGA, *BRAF*-reverse: 5'-TAG TAA CTC AGC AGC ATC TCA GGG C.

2 µg of gDNA were subjected to bisulfite conversion using the EpiTect Bisulfite Kit (Qiagen) according to the manufacturer's instructions. TaqMan-based methylation specific quantitative real-time PCR (MethyLight) was used to determine the CIMP-status. 8 CIMP-specific markers (CACNA1G, IGF2, NEUROG1, RUNX3, SOCS1, MLH1, CRABP1, CDKN2A) and a methylation-independent normalization control (ALU) were included in the analysis. Primer/probe sequences and protocol were described previously (De Sousa E Melo et al, 2013). Markers with a PMR value > 10 were considered positive for methylation; samples were defined as CIMP high (≥ 6 out of 8 CIMP markers have a PMR > 10), low (1-5 out of 8 CIMP markers have a PMR > 10), or negative (0 markers have a PMR > 10).

Appendix figures

Appendix Figure S1

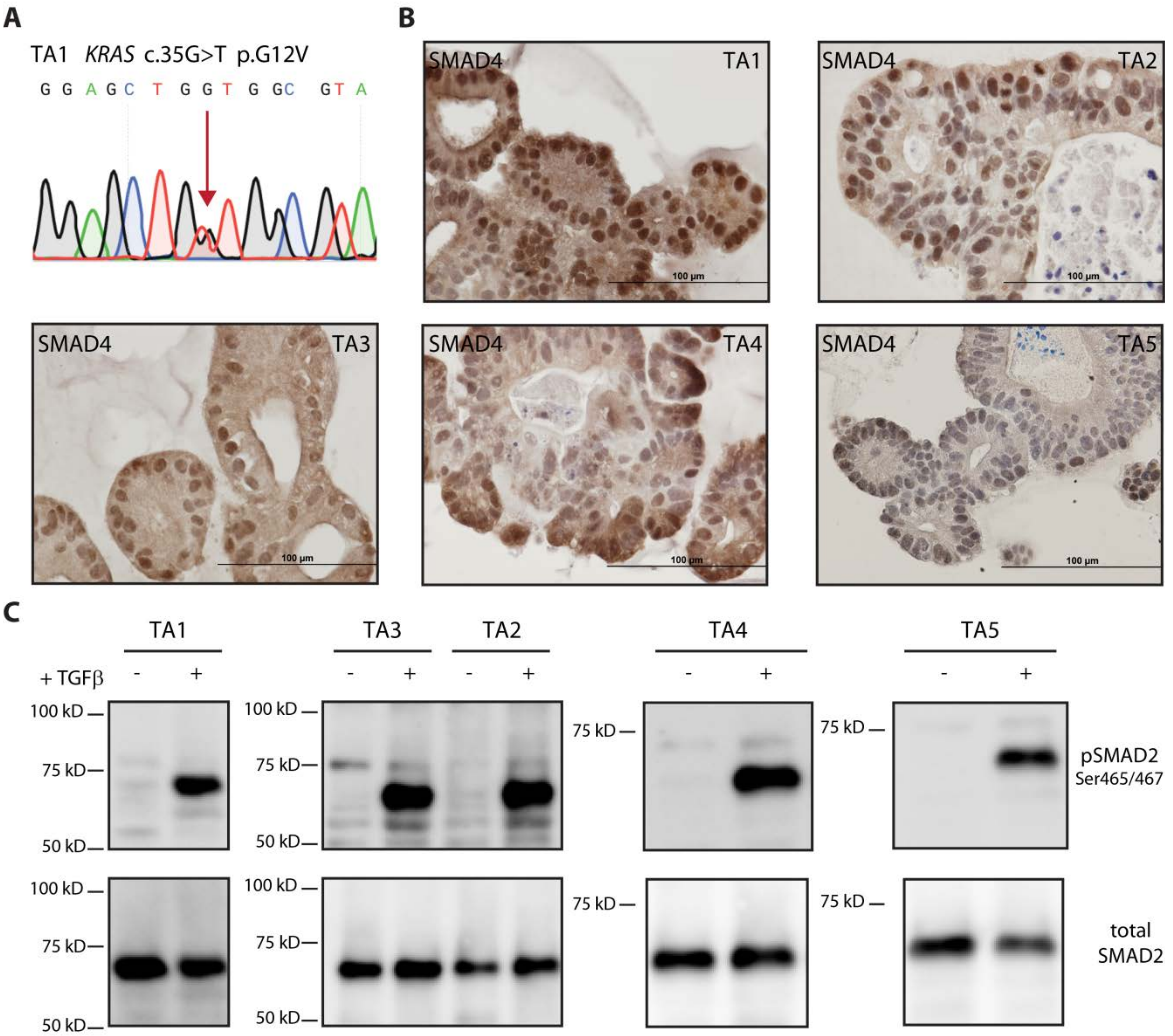

Appendix Figure S1. The TGFβ pathway is intact in TA organoid cultures.

A The TA1 organoid culture carries a *KRAS*^{G12V} mutation determined by Sanger sequencing.

B All five TA organoid cultures display SMAD4 expression.

C The TA organoids respond to TGFβ treatment with induction of phospho-SMAD2 (pSMAD2) levels.

Appendix Figure S2

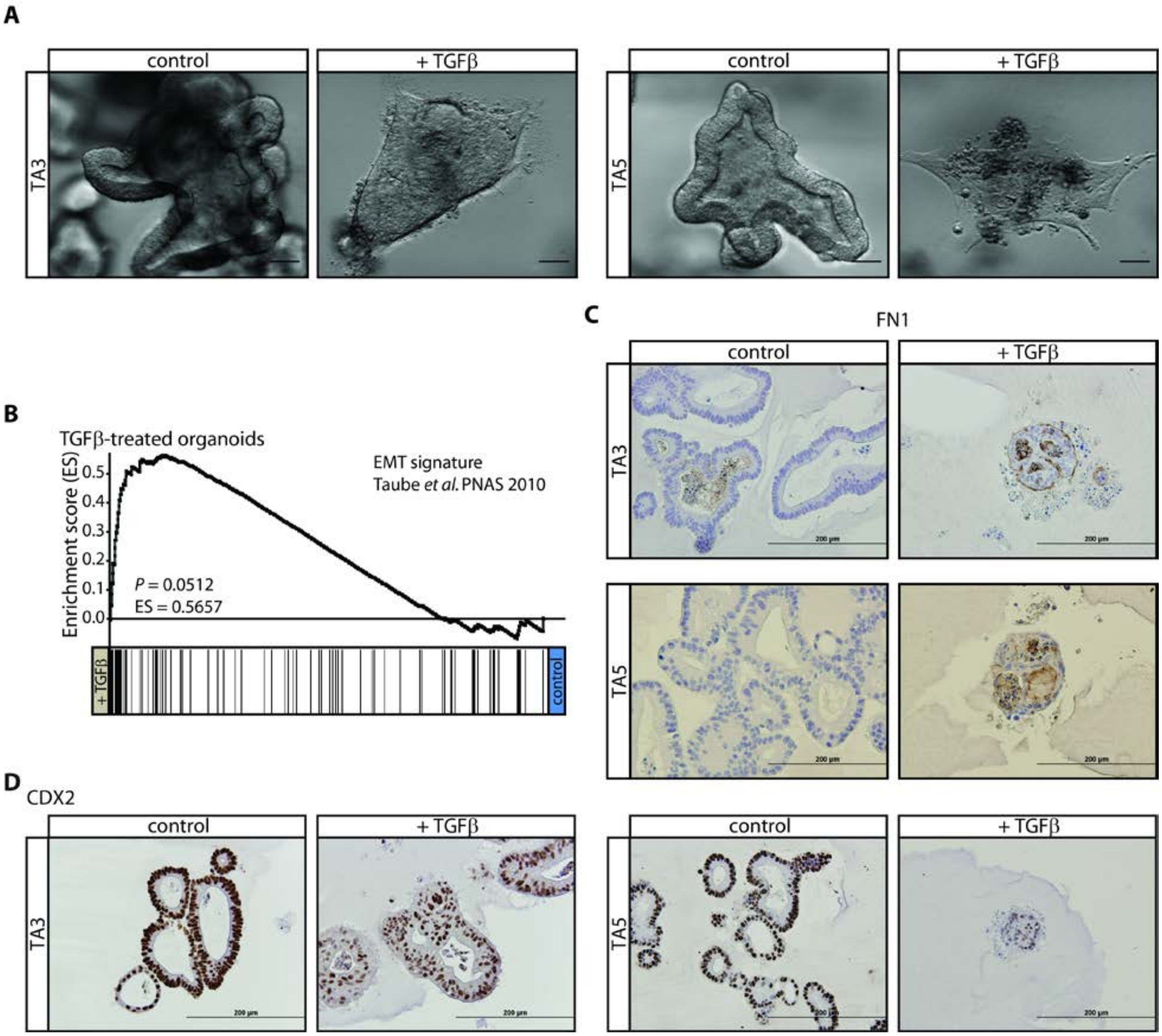

Appendix Figure S2. TGFβ stimulation induces EMT features in TA organoid cultures.

A Morphological changes in surviving TA organoids upon TGFβ treatment resemble the induction of a mesenchymal phenotype (scale bars: 50 μm).

B Genes present in an experimentally-derived EMT signature (Taube *et al.* 2010) are enriched in TGFβ-treated compared to control samples.

C The mesenchymal marker fibronectin 1 (FN1) is induced upon TGFβ treatment.

D CDX2 expression is reduced in TGFβ-treated TA organoid cultures.

Appendix Figure S3

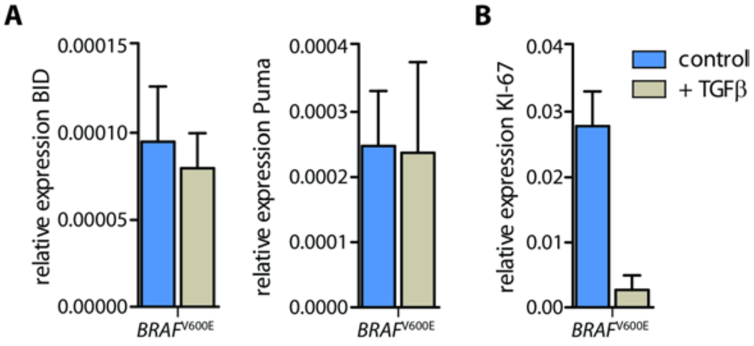

Appendix Figure S3. TGFβ induces growth arrest in genetically engineered *BRAF*^{V600E}-mutant organoids.

A The expression of the pro-apoptotic molecules BID and Puma (*BBC3*) are not changed upon TGFβ treatment in *BRAF*^{V600E}-mutated organoid cultures (n = 6).

B *BRAF*^{V600E}-mutant organoids undergo growth arrest in the TGFβ-treated condition judged by KI-67 downregulation (n = 6).
