# Supplementary material for: TGFβ signaling directs serrated adenomas to the mesenchymal colorectal cancer subtype
Source: EMBO Mol Med. 2016 May 24;8(7):745–60. doi: 10.15252/emmm.201606184 (PMC4931289; doi:10.15252/emmm.201606184)
Supplement: Supplementary file 2 — Expanded View Figures PDF [file EMMM-8-745-s002.pdf]

Expanded View Figures

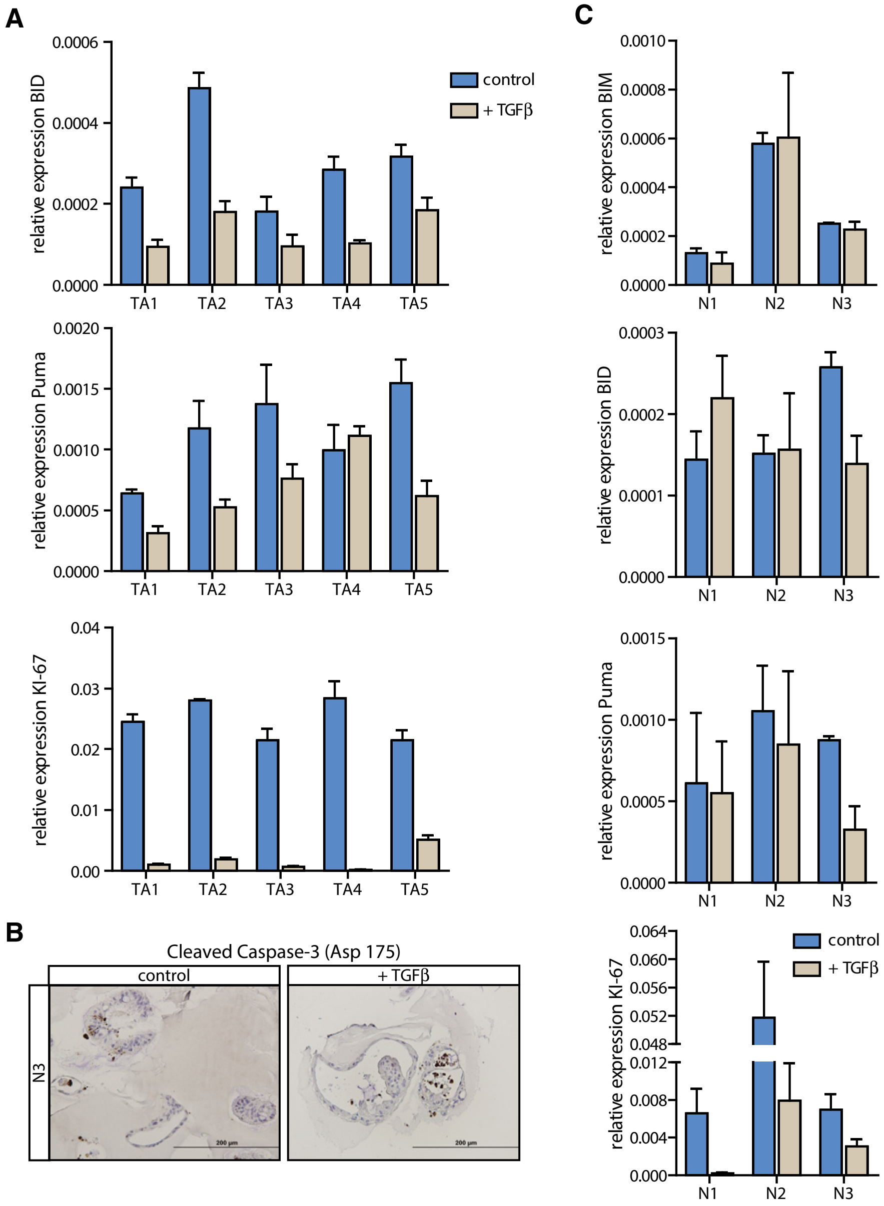

Figure EV1. TA and normal colon organoids display growth arrest upon TGFβ treatment, but normal organoids do not show features of apoptosis induction.

- A The pro-apoptotic molecules BID and Puma (*BBC3*) are not induced in TGFβ-treated TA organoids, but KI-67 is downregulated (one representative of ≥ 3 independent experiments is shown, error bars represent SD).
- B Cleaved Caspase-3 was not induced upon TGFβ stimulation in the normal colon organoid culture N3 (scale bars: 200 μm).
- C No induction of pro-apoptotic molecules was detected in TGFβ-treated normal colon cultures (N1-N3), but KI-67 expression was reduced [one (representative) experiment is shown ($n = 1$ for N1 and N2, and $n = 3$ for N3), error bars represent SD].

© 2016 The Authors

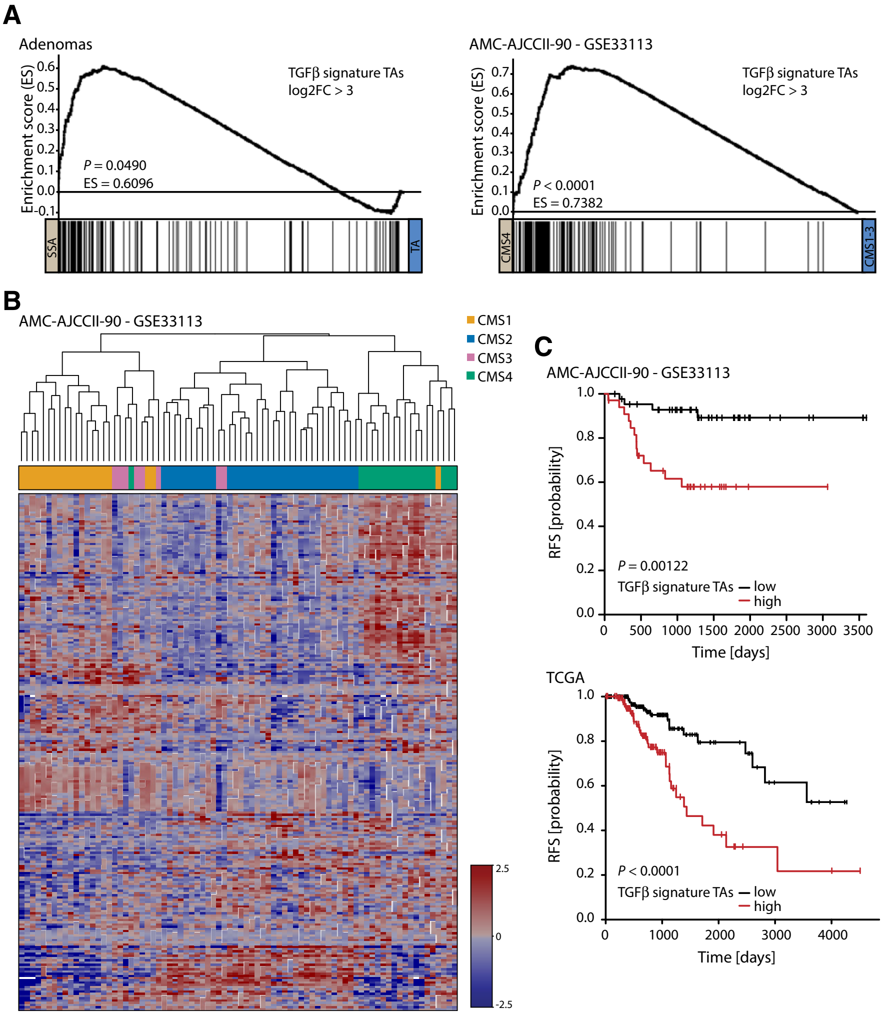

Figure EV2. Genes induced upon TGFβ treatment are enriched in CMS4 and SSA samples and a TGFβ signature is predictive of prognosis in two CRC datasets.

- A Genes induced in TGFβ-treated TA organoid cultures (log2FC > 3) are enriched in SSA compared to TA precursor lesions (n = 12 for TA and n = 15 for SSA; left) and in CMS4 versus CMS1-3 tumor samples of the AMC-AJCCII-90 dataset (CMS4 n = 20 and CMS1-3 n = 60; GSE33113; right).
- B A TGFβ signature derived from the stimulation of TA organoid cultures (|log2FC| > 3) is able to separate CMS4 from CMS1-3 tumors of the AMC-AJCCII-90 dataset. Expression values were mean-centered (genewise), and cosine similarity was used as the distance measure.
- C Separating patients into a TGFβ-low (black) or TGFβ-high (red) group based on the TGFβ signature derived from the stimulation of TA organoid cultures (|log2FC| > 3) reveals its prognostic value in two independent CRC patient datasets (*P*-values are based on log-rank tests; RFS—recurrence-free survival).

Source data are available online for this figure.

Figure EV3. The TGFβ target gene ZEB1 is expressed in SSAs and these precursor lesions are classified as either CMS1- or CMS4-like based on gene expression.

- A The TGFβ target gene ZEB1 is highly expressed in epithelial cells of SSA but not of TA precursor lesions (scale bars: 200 μm).
- B Classification of adenoma samples (n = 12 for SSA and n = 15 for TA) into one of the CMSs based on gene expression (RF—random forest).
- C Mutation analysis of adenoma samples of which gene expression data were derived: CIMP status (white: CIMP negative, gray: CIMP low, black: CIMP high), *BRAF* mutation (white: $BRAF^{wildtype}$, black: $BRAF^{V600E}$), and *KRAS* mutation (white: $KRAS^{wildtype}$; all samples analyzed displayed the wild-type sequence for codon 12 and codon 13 of exon 2 and codon 61 of exon 3 of the *KRAS* gene). Asterisks indicate data not available (n.a.).

 © 2016 The Authors

A

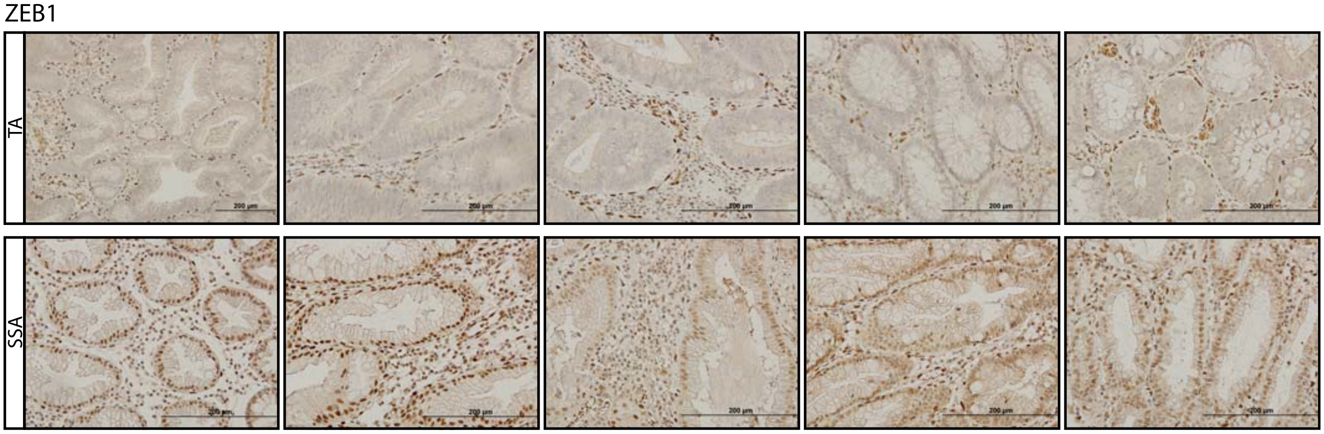

B

adenoma samples	RF CMS1 posterior probability	RF CMS2 posterior probability	RF CMS3 posterior probability	RF CMS4 posterior probability	predicted CMS
**SSA2**	0.72	0.02	0.07	0.19	1
**SSA3**	0.08	0.00	0.02	0.90	4
**SSA4**	0.34	0.04	0.04	0.58	4
**SSA5**	0.23	0.02	0.03	0.72	4
**SSA6**	0.53	0.11	0.20	0.16	1
**SSA9**	0.61	0.11	0.10	0.18	1
**SSA10**	0.55	0.14	0.11	0.20	1
**SSA12**	0.57	0.14	0.26	0.03	1
**SSA13**	0.25	0.04	0.01	0.70	4
**SSA14**	0.57	0.08	0.07	0.28	1
**SSA15**	0.51	0.05	0.07	0.37	1
**SSA17**	0.70	0.04	0.11	0.15	1
**TA2**	0.04	0.50	0.44	0.02	2
**TA3**	0.04	0.35	0.60	0.01	3
**TA4**	0.09	0.64	0.25	0.02	2
**TA5**	0.05	0.73	0.22	0.00	2
**TA6**	0.09	0.62	0.20	0.09	2
**TA7**	0.11	0.60	0.22	0.07	2
**TA8**	0.10	0.54	0.27	0.09	2
**TA9**	0.06	0.54	0.38	0.02	2
**TA10**	0.07	0.57	0.35	0.01	2
**TA11**	0.04	0.69	0.24	0.03	2
**TA12**	0.12	0.57	0.24	0.07	2
**TA13**	0.11	0.61	0.20	0.08	2
**TA14**	0.10	0.61	0.20	0.09	2
**TA15**	0.10	0.52	0.29	0.09	2
**TA16**	0.12	0.32	0.55	0.01	3

C

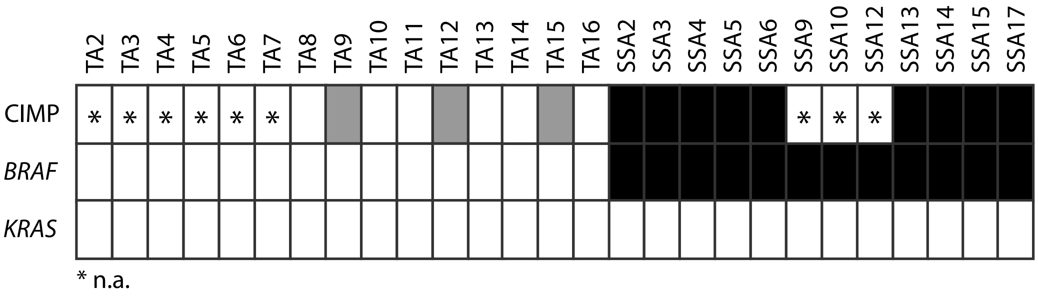

Figure EV3.

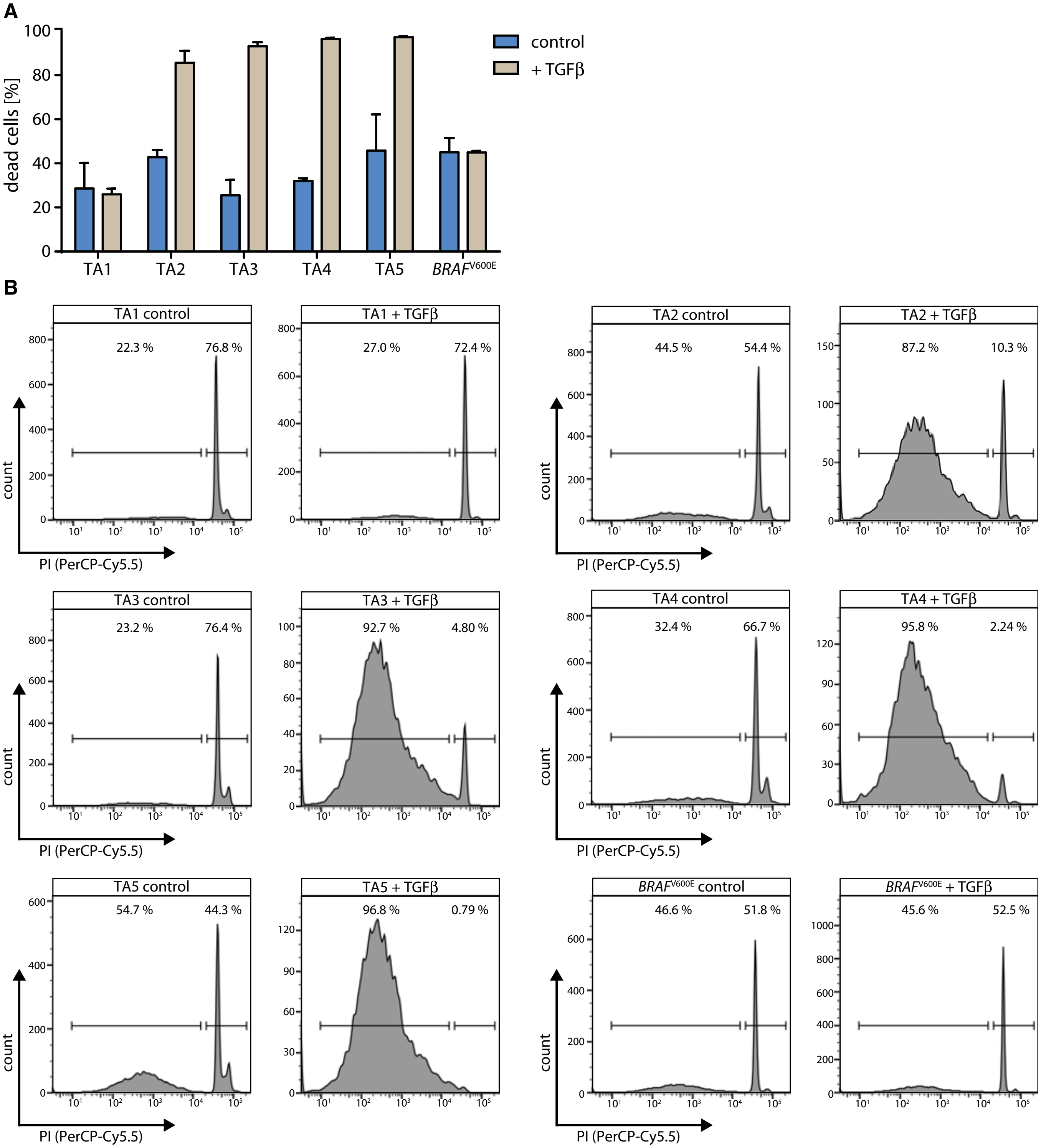

Figure EV4. Cell death is the dominating response to TGFβ treatment in *KRAS*-wild-type TA organoid cultures, but is not induced in the *BRAF*^{V600E}-mutant organoid culture.

A Displayed is the percentage of dead cells in the control or TGFβ-treated condition after 5 days determined by Nicoletti assay (one representative of ≥ 3 independent experiments is shown, error bars represent SD). To control for differences in medium composition and to rule out a protective effect of the normal colon culture medium, the *BRAF*^{V600E}-mutant organoid culture was plated in TA culture medium for the duration of this experiment (5 days).

B One representative FACS plot of the control and TGFβ-treated sample is shown for each organoid culture.

 © 2016 The Authors

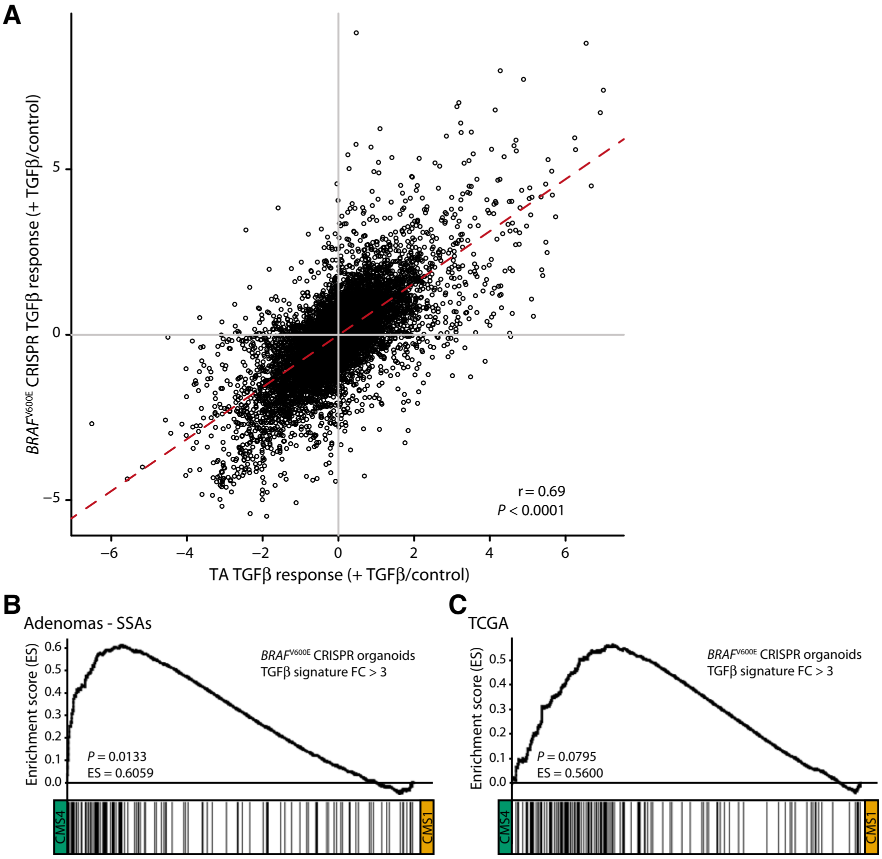

Figure EV5. Genes regulated by TGFβ in the *BRAF*^{V600E}-mutant organoid culture display differential expression in CMS4 versus CMS1 tumors.

- A Correspondence of the TGFβ response in the *BRAF*^{V600E}-mutant organoid culture and TA organoids. The red dotted line is the regression line; *r* = Pearson correlation coefficient; *P*-value is based on a Pearson's correlation test.
- B Genes induced by TGFβ treatment of the *BRAF*^{V600E}-mutant organoid culture are enriched in CMS4-SSA compared with CMS1-SSA samples (*n* = 4 for CMS4-SSAs and *n* = 8 CMS1-SSAs).
- C TGFβ target genes upregulated upon TGFβ treatment of the *BRAF*^{V600E}-mutant organoid culture are enriched in *BRAF*-mutant CMS4 CRCs compared with *BRAF*-mutant CMS1 carcinomas of the TCGA dataset (*n* = 4 for CMS4 and *n* = 34 for CMS1 CRC samples).

Source data are available online for this figure.

© 2016 The Authors
