# Supplementary figures and images for: TGFβ signaling directs serrated adenomas to the mesenchymal colorectal cancer subtype
Source: EMBO Mol Med. 2016 May 24;8(7):745–60. doi: 10.15252/emmm.201606184 (PMC4931289; doi:10.15252/emmm.201606184)

Source Data Appendix Figure S1

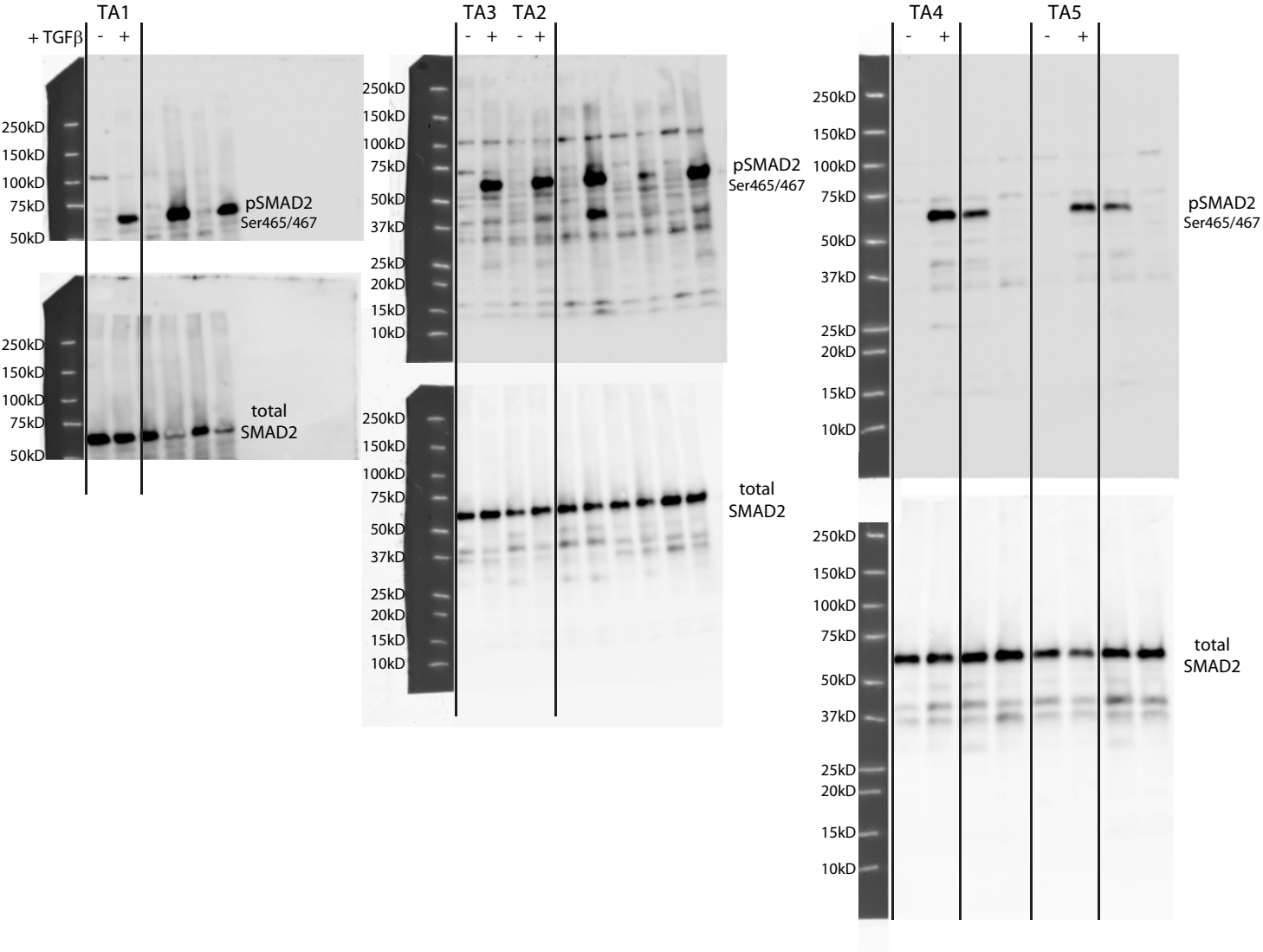

Supplement: Supplementary file 3 — Source Data for Expanded View and Appendix [file EMMM-8-745-s006.zip › Source_Source_data_for_Expanded_View_and_Appendix/Source_data_for_Appendix_Fig_S1.pdf]

Source Data Appendix Figure S2

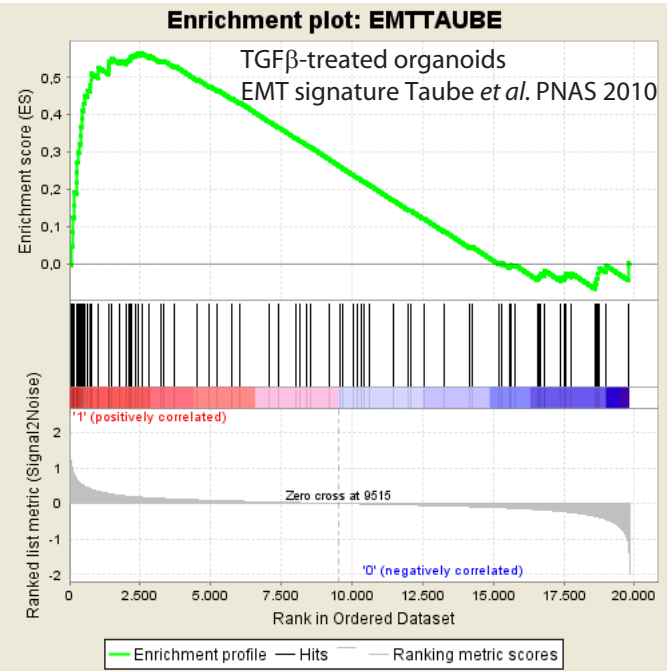

Supplement: Supplementary file 3 — Source Data for Expanded View and Appendix [file EMMM-8-745-s006.zip › Source_Source_data_for_Expanded_View_and_Appendix/Source_data_for_Appendix_Fig_S2.pdf]

Source Data Figure EV5

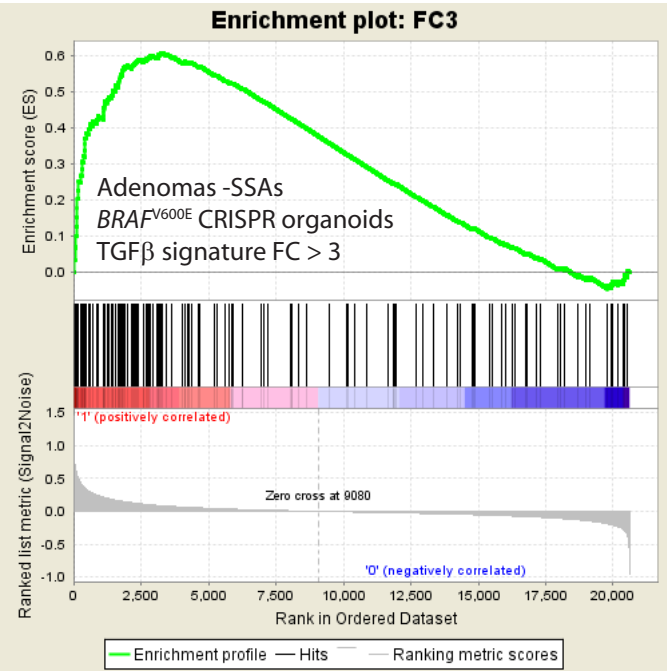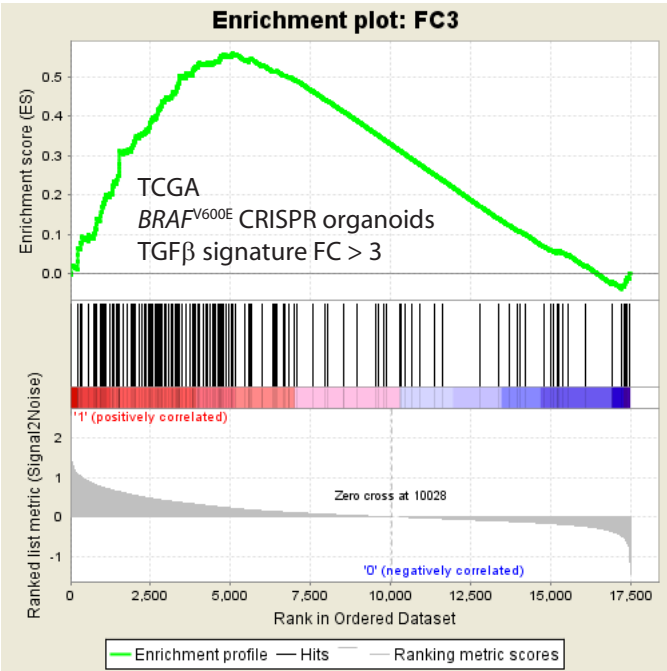

Supplement: Supplementary file 3 — Source Data for Expanded View and Appendix [file EMMM-8-745-s006.zip › Source_Source_data_for_Expanded_View_and_Appendix/Source_data_for_Fig_EV5.pdf]

Source Data Figure EV2

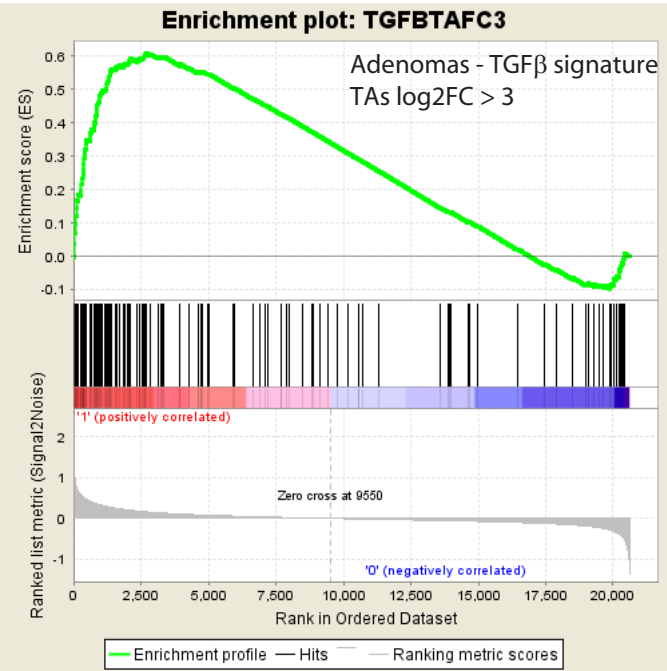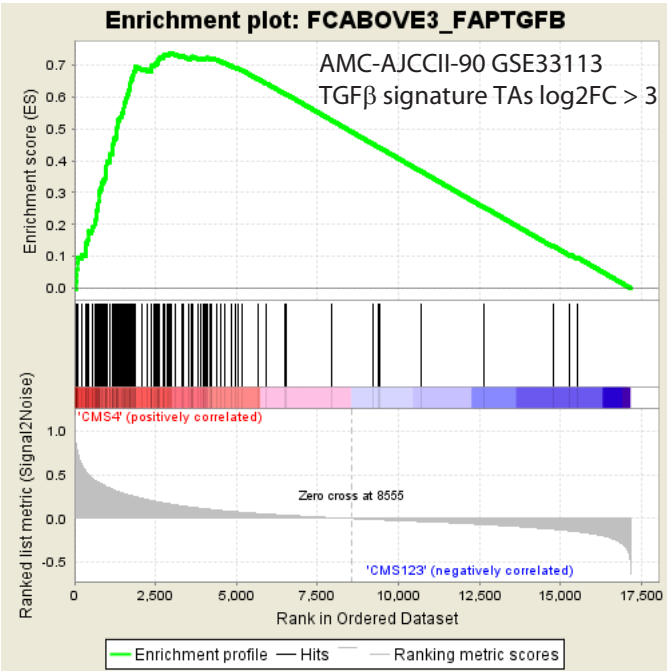

Supplement: Supplementary file 3 — Source Data for Expanded View and Appendix [file EMMM-8-745-s006.zip › Source_Source_data_for_Expanded_View_and_Appendix/Source_data_for_figure_EV2.pdf]

Source Data Figure 1

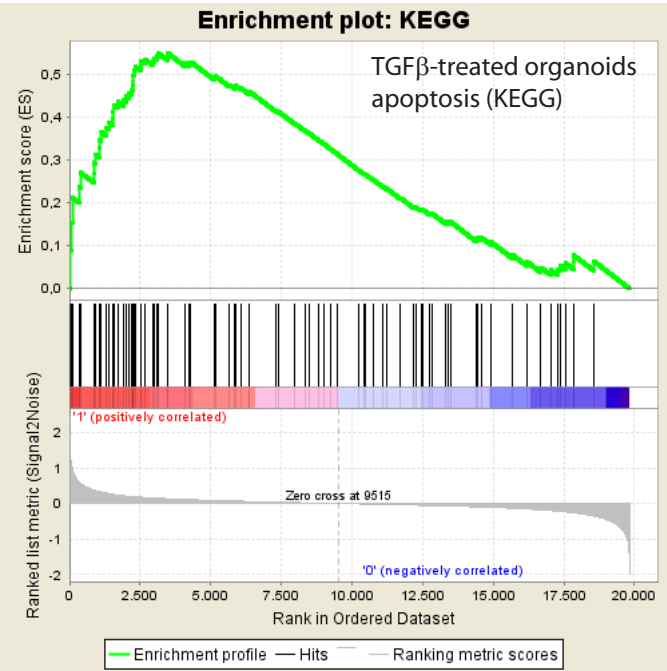

Supplement: Supplementary file 5 — Source Data for Figure 1 [file EMMM-8-745-s003.pdf]

Source Data Figure 2

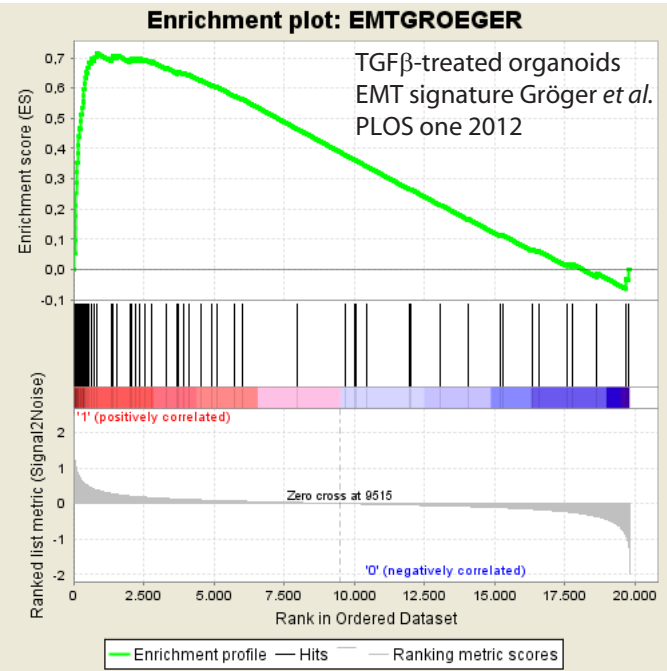

Supplement: Supplementary file 6 — Source Data for Figure 2 [file EMMM-8-745-s004.pdf]

Source Data Figure 3

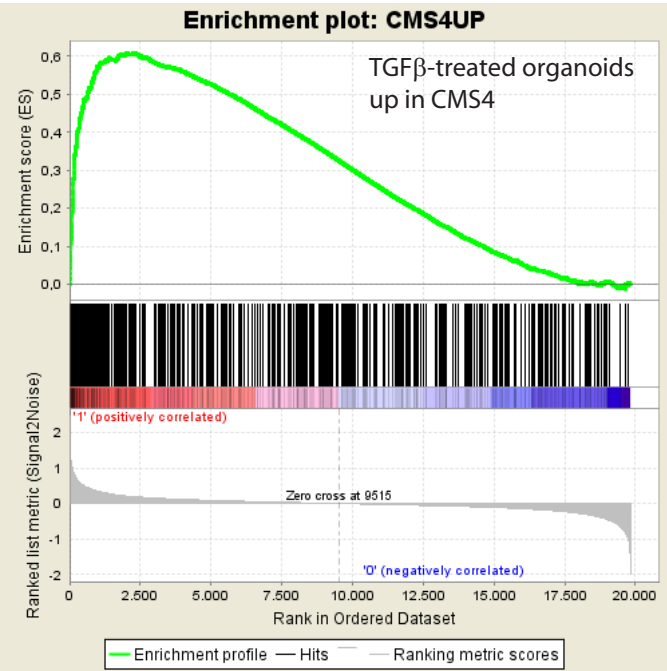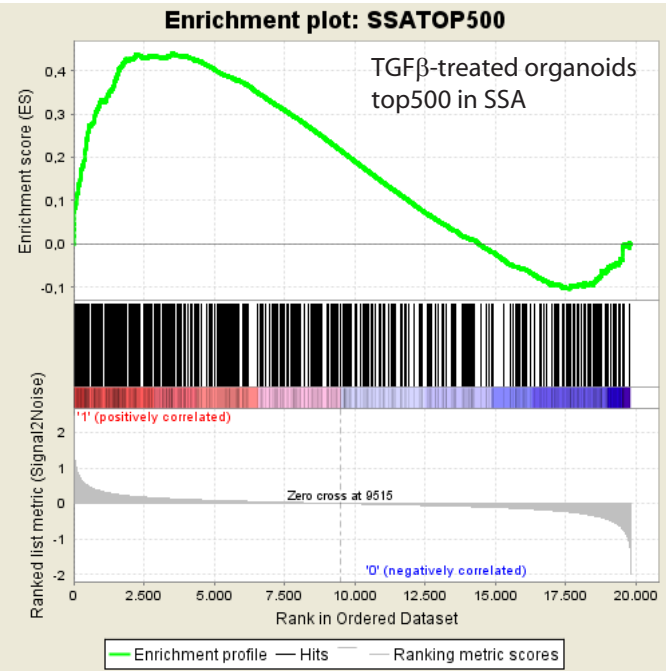

Supplement: Supplementary file 7 — Source Data for Figure 3 [file EMMM-8-745-s005.pdf]
